# Geometry evolution of mesoscopic mechanical structures during the rock fragmentation process induced by tunnel boring machine (TBM) cutters

**DOI:** 10.1098/rsos.211630

**Published:** 2022-01-26

**Authors:** Qi Geng, Fei He, Zhiyong Lu, Xiaohui Liu, Xuebin Wang, Min Ye

**Affiliations:** ^1^ National Engineering Laboratory for Highway Maintenance Equipment, School of Construction Machinery, Chang'an University, Xi'an 710064, People's Republic of China; ^2^ Post-Doctoral Research Center, Tibet Tianlu Co., Ltd, Lasa 850000, People's Republic of China; ^3^ China Railway Engineering Equipment Group Co., Ltd, Zhengzhou 450016, People's Republic of China

**Keywords:** tunnel boring machine, rock fragmentation, grain-based discrete element method, mesoscopic evolution, meso-loop

## Abstract

We investigated the geometric evolution of mesoscopic mechanical structures in rocks during the rock fragmentation process induced by tunnel boring machine cutters. Numerical models were built using a grain-based discrete element method to accurately represent the mesoscopic structures and macroscopic mechanical behaviours of rocks, and the relationship between the mesoscopic evolution and the macroscopic response of rock was determined. The major results are as follows. First, the crushing and re-compaction of the grains were found to mainly occur in the thin crushed zone immediately beneath the cutter tip. Second, the reduction in the bearing ability of the dense core during cutter indentation was due to the order increment of the contact topological structure at the mesoscopic scale. Third, the area percentages of low- and high-order meso-loops decreased and increased, respectively, during the indentation process, and the volume expansion of the dense core was mainly caused by an increase in the internal pore area of high-order meso-loops that have low internal solid fractions. Fourth, the low-order meso-loops primarily bore and transferred the indentation force. Finally, the distribution contour of the meso-loops was found to be an appropriate and intuitive approach for representing the evolution of cracks on a macroscopic scale.

## 1. Introduction

Full-face tunnel boring machines (TBMs) are major high-end equipment for rock tunnel excavation and are especially applied to large mountain tunnel projects and inter-basin water diversion works. The movements of the cutterhead system are described as follows: the cutterhead rotates and advances forward under the joint action of the driving and propulsion systems, resulting in the disc cutters installed on the cutterhead grinding on the tunnel face in concentric circles. Accordingly, the rock fragmentation mechanism can be demonstrated as follows: a high-pressure dense core forms beneath the cutter ring tip as the disc cutter penetrates the rock surface; this drives the propagation of the lateral and median cracks that initiate from the side of the dense core and their connection with cracks produced by neighbouring cutters, generating flat or elongated rock chips.

Investigation of the rock fragmentation mechanism is of vital importance as it directly affects the design of cutter-cutterhead systems and the selection of TBM operation parameters. During the past two decades, owing to the rapid advancement of numerical simulation technologies and computing power, several numerical studies have been conducted to investigate the rock fragmentation process induced by TBM cutters. The representative studies are summarized in table 1.

**Table 1.** Summary of the representative numerical studies on the rock fragmentation process induced by TBM cutters during the past two decades (FEM, finite-element method; DEM, discrete element method; SPH, smoothed particle hydrodynamics; VE-NMM, Voronoi element-based numerical manifold method).

method and software	main concerns, and the studied parameters	resources
FEM, RFPA^2D^	fracturing and indentation force; confining stress and cutter spacing	Liu *et al*. [1]
DEM, UDEC	fracturing, crack mode and stress field; joint orientation and spacing	Gong *et al*. [2,3]
Lagrangian element method, FLAC^2D^	indentation force, groove dimension, fracture mode and type; lateral confinement, free face spacing, rock types	Innaurato *et al*. [4]
DEM, PFC^2D^	fracturing process, crack distribution; cutter ring shape, rock strength	Su *et al*. [5]
DEM, UDEC	fracturing process, stress contour, cracks; spherical tooth hob cutter	Tan *et al*. [6]
FEM, AUTODYN^3D^	fracturing result, rolling force, specific energy; penetration, spacing	Cho & Jeon [7,8]
DEM, PFC^2D^	cracks, chips; cutter spacing, joint strength, orientation and spacing	Sun *et al*. [9]
FEM, RFPA^2D^	indentation force, fragmentation, crack, energy; confining stress, ratio	Ma *et al*. [10]
DEM, UDEC	fracturing, crack; cutter ring shape, rock strength, joint orientation	Mo *et al*. [11]
DEM, PFC^2D^	fracturing process, penetration force, side force, crack, specific energy; cutter spacing and penetration	Tan *et al*. [12,13]
DEM, PFC^2D^	indentation force and specific energy; cutter spacing and penetration	Moon & Oh [14]
DEM, UDEC	fracturing process, crack length, crushed zone depth; confining stress	Peng [15]
DEM, PFC^3D^	rock cutting forces, specific energy; cutter spacing and penetration	Choi & Lee [16]
DEM, PFC^2D^	dynamic fracturing process, cracks; impact frequency and velocity	Liu *et al*. [17]
FEM, ABAQUS^3D^	DP and MC model, VUMAT, thrust, torque; penetration and UCS	Han *et al*. [18,19]
FEM, LS-DYNA^3D^	lateral cutting force, cutter wear and rock damage; penetration depth	Yang *et al*. [20]
DEM/FEM coupling, DEMpack	rock cutting forces, cutting coefficient, specific energy; cutting modes, cutter geometry, cutting velocity, cutter penetration and spacing	Labra *et al*. [21]
DEM, PFC^2D^	GBM method, cracks, indentation force, porosity, stress, fragment efficiency, penetration rate; wedge angle, confining stress, etc.	Li *et al*. [22]
FEM, ABAQUS^3D^	DP model, VUMAT, cutter force, specific energy; cutter spacing	Geng *et al*. [23,24]
FEM, LS-DYNA^3D^	rock fragmentation, rolling force, specific energy; free face spacing	Xia *et al*. [25]
SPH/FEM coupling, ABAQUS^3D^	cracks, rock cutting forces; numerical and experimental comparison	Xiao *et al*. [26]
VE-NMM^2D^ model	rock fragmentation, cracks, indentation load; confining stress	Liu *et al*. [27]
DEM, PFC^2D^	cracks, indentation force; mixed rock and different interface angle	Zhang *et al*. [28]
DEM, PFC^3D^	cracks, rolling forces, specific energy; mixed ground, penetration	Zhang *et al*. [29]
DEM, PFC^2D^	GBM method, cracks, stress, indentation force; free face conditions	Xu *et al*. [30]

It was found that the most applied numerical methods in these papers are the finite-element method (FEM) and discrete element method (DEM), which account for approximately 40% and 60% of the applied numerical methods, respectively. Focusing on the critical problem of accurate representation of rock material, numerical models have been continuously improved. First, many FEM-based rock-cutting simulations regard the rock as an isotropic material and represent the rock failure process using an element deletion approach controlled by a damage factor assigned to the elastic modulus. Thus, the critical rock re-compaction behaviour in the crushed zone immediately beneath the disc cutter cannot be well represented; as a result, the rock chipping performance and rock cutting forces cannot be reasonably obtained [24]. To overcome these shortcomings, some researchers have explored DEM/FEM coupling [21] or smoothed particle hydrodynamics/FEM coupling [26] approaches by representing a crushed zone with discrete particles and other parts with meshes. Second, compared with the FEM-based numerical approaches, a significant advantage of the DEM-based approaches, for example, the particle flow code (PFC), is that they provide visual representations of crack initiation and propagation. However, their simulation accuracy is usually restrained by the low rolling resistance between neighbouring spherical particles if the linear parallel bond contact model is directly assigned [31]. To solve this problem, some grain-based methods (GBM) [22,27,30,32] have been developed by grouping the particles into breakable clusters or unbreakable clumps according to the size and shape of the mineral grains; hence, the rock chipping behaviour and rock cutting forces can be accurately simulated.

It was also found that these numerical simulations mainly focus on the macroscopic scale, considering rock cutting performance indicators such as rock indentation or cutting forces, rock fragmentation or fracturing performance, macro crack evolution and rock cutting efficiency. This suggests that the rock cutting performance under different conditions considering rock materials, cutter geometries and cutting parameters can be well evaluated, which can help design the TBM cutterhead and select the operation parameters. However, investigations on the mesoscopic mechanical behaviours of the rock fragmentation process induced by TBM cutters are rare. In recent years, research on the multi-scale mechanical behaviour of geotechnical granular materials shows that the macroscopic response of materials under an external load is closely related to the evolution of the particle contact structure at the mesoscopic scale [33]. Herein, the representative studies are listed as follows: Qin & Chi [34] simulated the biaxial shear process of granular material using the software PFC^2D^ and discussed the relationship between the volume change of void cells and the shape evolutions of void cells; Zhu *et al*. [35,36] simulated the drained biaxial test of granular material using the DEM software YADE and analysed the critical state fabric in different failure modes on a mesoscopic scale considering force chains and meso-loops; Liu *et al*. [37–39] simulated the biaxial compression and biaxial shear tests of granular material using the software PFC^2D^, and analysed the evolution characteristics of meso-mechanical structures such as porosity, coordination number and loop element; Ling *et al*. [33] simulated the biaxial compression process of soil–rock mixtures under flexible boundaries using the software PFC^2D^ and investigated the evolution of the micro-contact structure in the internal shear bands and its influence on the macroscopic deformation characteristics. These very interesting studies took advantage of the DEM method in investigating the mechanical behaviours of granular materials at the microscopic and mesoscopic scales, and inspired us to explore the evolution of mesoscopic indicators during the rock fragmentation process induced by TBM cutters, which may provide new insights into the evolution of the crushed zone and dense core.

The major objective of this study is to investigate the geometric evolution of the mesoscopic mechanical structure in the rock fragmentation process induced by TBM cutters. In §2, two-dimensional rock indentation models were built considering the constant cross-section (CCS)- and V-type disc cutters. As many studies (e.g. [3,4,22]) have verified the appropriateness of the two-dimensional equivalent model, disc cutter indentation was considered a plane problem, and tangential rolling was ignored. The rock sample was synthesized using a GB-DEM, where the assembled polycrystalline clusters were breakable from both grain bodies and grain boundaries. In §3, the rock indentation processes were analysed considering the macroscopic rock fragmentation performance and cutter indentation force. In §4, the evolution of the coordination number was first analysed during the entire process of cutter indentation. Then, the organizations among the particle contacts were characterized by the topology of the meso-structures called loops, and the evolution of the number, area and shape of the loops in the crushed zone, macro crack zone and other parts of the rock sample were analysed. Finally, the influence of particle packing parameters on mesoscopic structures was studied.

## 2. Set-up of the numerical models

### 2.1. Modelling of the rock sample using the GB-DEM approach

**Figure 1.**
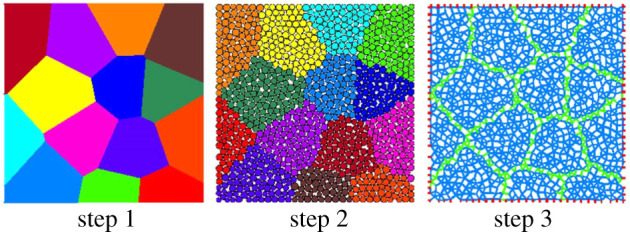
Illustration of the GB-DEM modelling process using a Voronoi polygon algorithm.

The linear parallel bond model written as ‘linearpbond’ in PFC is a commonly used contact model for rocks, where neighbouring particles are connected and locked with parallel bonds that can be envisioned as elastic springs with limited shear and tensile strengths as well as rotation constraints [40]. However, it has been indicated that, under uniaxial loading, the ratio of compressive strength to tensile strength of synthesized rocks is obviously lower than that of real rocks when the spherical particles are directly connected via parallel bonds [31]. Such a discrepancy may be caused by the lack of interlocking friction along the irregular rock grain boundaries, which are approximated as smooth surfaces, in the spherical particle aggregate [22]. In this study, a modified Voronoi polygon algorithm was used to group the particles into grains to represent polycrystalline rocks and to avoid the uncontrolled randomness of the shape and size of grains generated using primary Voronoi polygons. As depicted in figure 1, the GB-DEM approach for modelling a synthesized rock comprises three steps. During the first step, the rock domain is filled with Voronoi polygons in three sub-steps. In sub-step 1, the rock domain is divided into Delaunay triangles. In sub-step 2, the centres of the triangles' circumferential circles are connected around the triangle vertices. In sub-step 3, the Delaunay triangles are deleted, leaving behind the Voronoi polygons. During the second step, the grains are generated based on the Voronoi polygons created in the first step. In sub-step 4, the rock domain is filled with spherical particles; in sub-step 5, the particles are divided into groups based on the Voronoi polygons, meaning that the particles that fall into the same Voronoi polygon share the same group name. During the third step, the parameters of the intragranular and intergranular contacts are calibrated and assigned. In sub-step 6, the linear parallel bond model is assigned to the contacts between the particles, and the linear model is assigned to the contacts between the particles and walls; in sub-step 7, the particle contact parameters are calibrated and assigned.

**Figure 2.**
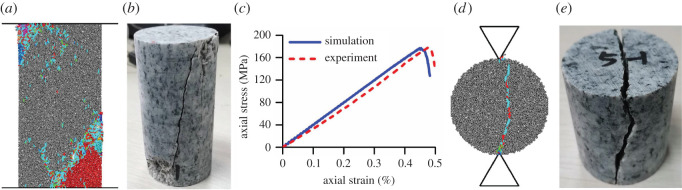
Simulations and experiments used for the calibration of microscopic properties: (*a*) simulation of the UCS test; (*b*) the physical UCS test; (*c*) curves of axial stress versus strain during the UCS test and simulation; (*d*) simulation of the BTS test; (*d*) the physical BTS test (note: the simulations were conducted on the sample-0 whose properties and parameters were given in tables 2 and 3).

The particle contact parameters were calibrated according to the following steps. First, a series of uniaxial compression strength (UCS) and Brazilian tensile strength (BTS) tests were conducted on granite collected from the Qinling Mountains, Shaanxi, China, according to the testing method suggested by the International Society of Rock Mechanics (ISRM), to obtain the macroscopic mechanical properties listed in table 2. Second, groups of UCS and BTS tests were simulated for the rock synthesized using the approach described in the preceding paragraph. To ensure that the sample remained in a quasi-static equilibrium throughout the UCS and BTS tests, the loading rate was set to 0.1 m s^−1^ and the time step was approximately 5.5 × 10^−8^ s according to Cho *et al*. [31]. The calibration procedure was basically a trial-and-error process and the termination condition was that the errors between the Young's modulus, Poisson's ratio, UCS and BTS values of the synthesized rock sample and those of the collected rock specimen were smaller than 5%. If so, the selected microscopic parameters were considered suitable. To reduce the number of independent parameters, several assumptions were made for the calibration procedure according to Li *et al*. [32]: (i) the density of the particles was determined by the realistic density of the rock material, (ii) the Young's modulus was determined by the effective modulus and the Poisson's ratio was determined by the stiffness ratio, (iii) the effective modulus of the particles and bonds was set the same and so was the normal-to-shear stiffness ratio, and (iv) the packing parameters including the particle radius, particle porosity and grain size were selected based on experience and previous studies [22,31,40]. As noted in the sub-step 5 of the preceding paragraph, the particles falling into the same Voronoi polygons share the same group name. It indicates that the two particles connected by the contact within a grain have the same group name, while the two particles connected by the contact in a grain boundary have different group names. This is the basic logic to distinguish the contacts in the grain boundaries and within the grains when assigning the contact parameters. Using the microscopic properties shown in table 3, the synthesized granite sample presents very similar failure patterns and macroscopic mechanical behaviours to the collected granite specimen (table 2 and figure 2), which indicates that the numerical method is reliable. The granite was crushed so that it could fail along an inclined fracture surface during the UCS experiment and simulation, and it was split into two intact pieces along the centre surface during the BTS experiment and simulation. Furthermore, the curves of the axial stress versus strain during the UCS experiment and simulation were close to each other. Moreover, the errors between the Young's modulus, Poisson's ratio, UCS and BTS of the synthesized granite sample and the collected granite specimen were all lower than 4%. Four types of cracks, that is, intergranular tensile cracks, intragranular tensile cracks, intergranular shear cracks and intragranular shear cracks, are illustrated in cyan, red, green and blue colours, respectively. During the UCS test simulation, the tensile cracks and intergranular tensile cracks accounted for approximately 84% and 61% of all the cracks, respectively. During the BTS test simulation, the tensile cracks and intergranular tensile cracks accounted for approximately 86% and 56% of all the cracks, respectively. This means that the major rock failure pattern is tensile failure of the contacts between neighbouring particles in neighbouring grains.

**Table 2.** Mechanical properties of the synthesized rock samples for simulation and the collected rock specimen for experiment (note: sample-0 is for §3 and §§4.1–4.6, and sample-1 and -2 are for §4.7 of sensitivity analysis).

rock	Young's modulus, *E* (GPa)	Poisson's ratio, *υ*	UCS (MPa)	BTS (MPa)
collected specimen	38.6	0.170	177.9	12.3
synthesized sample-0	40.1	0.1697	176.8	12.0
relative error (%)	3.9	0.2	0.6	2.4
synthesized sample-1	37.4	0.168	176.2	12.7
relative error (%)	3.1	1.2	1.0	3.3
synthesized sample-2	38.9	0.171	181.0	12.6
relative error (%)	0.8	0.6	1.7	2.4

**Table 3.** Microscopic parameters of particles and bonds (note: sample-0 is for §3 and §§4.1–4.6, and sample-1 and -2 are for §4.7 of sensitivity analysis).

parameters		sample-0	sample-1	sample-2
*particle-based material parameters*				
particle density (kg m^−3^)		2610	2610	2610
particle radius (mm)		0.4–0.6	0.4–0.8	0.5–0.83
porosity		0.1	0.13	0.15
effective modulus, *emod* (GPa)		20	21	24
normal-to-shear stiffness ratio, *kratio*		1.2	1	1.2
average grain size (mm^2^)		3.2	6.4	4.8
*bond-based material parameters*				
effective modulus, *pb*_*emod* (GPa)		20	21	24
normal-to-shear stiffness ratio, *pb*_*kratio*		1.2	1	1.2
tensile strength, *pb_ten* (MPa)	intragranular	90 ± 9	150 ± 15	200 ± 20
	intergranular	70 ± 7	150 ± 15	200 ± 20
cohesion, *pb_coh* (MPa)	intragranular	90 ± 9	90 ± 9	90 ± 9
	intergranular	90 ± 9	100 ± 10	120 ± 12
friction coefficient		0.5	0.5	0.5

### 2.2. Rock indentation numerical models

**Figure 3.**
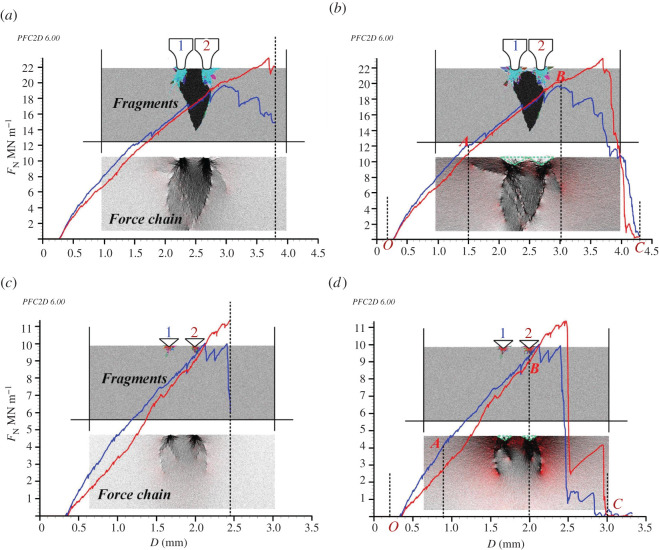
Rock indentation performances with CCS- and V-type disc cutters: (*a*) CCS model, *D* = 3.8 mm; (*b*) CCS model, *D* = 4.3 mm; (*c*) V model, *D* = 2.45 mm; (*d*) V model, *D* = 3.3 mm. (Note: the *F*_N_–*D* curves of cutter 1 and 2 are coloured in blue and red, respectively; the compression and tension force chains are coloured in black and red, respectively.) ©2020 Itasca consulting group, Inc.

The rock indentation numerical model is shown in figure 3. The tip width of the CCS-type cutter was 20 mm, the wedge angle of the V-type cutter was 100°, and the cutter spacing was 70 mm for both models. The length and width of the rock samples were 500 mm and 200 mm, respectively. The rock sample was composed of 113 055 particles that were grouped into 31 352 grains. The microscopic properties have been calibrated in §2.1 and are shown in table 3. The cutter indentation speed was set as 0.2 m s^−1^ (time step is 6.0 × 10^−8^ s) to ensure the quasi-static equilibrium of the rock sample. The initial distance between the ring tip and the top surface of the rock sample was set as 0.3 mm. The indentation process was terminated when the indentation force dropped to approximately 0.

## 3. Macroscopic rock indentation performance

The macroscopic rock indentation performances of the CCS- and V-type cutters are shown in figure 3. According to the curves of the cutter indentation force (*F*_N_) versus indentation depth (*D*), the indentation process includes two stages: loading and unloading. Note that *D* equals (*D*_N_ + 0.3) and *D*_N_ is the net indentation depth. As shown in figure 3*a,b*, during the loading stage, the *F*_N_ of CCS-type cutter 1 and 2 increased linearly to their peak values of 1.95 × 10^7^ and 2.32 × 10^7^ N m^−1^, respectively, at *D* of 3.0 and 3.7 mm, respectively. During the unloading stage, the *F*_N_ of the two cutters dropped rapidly to approximately 0 when *D* is 4.3 mm, which is called the critical indentation depth (*D*_C_). There were many small fluctuations in both the loading and unloading stages of the curves. The change in *F*_N_ with *D* was consistent with the jump crashing of rocks [12,14,41]. The rock beneath the cutters was compressed into many small fragments in the loading stage, and these fragments acted as a dense core to further bear and transfer the indentation force into the rock and drive the macro cracks to propagate. These results are similar to those of previous simulations [1,27,28]. As shown in figure 3*a,b*, the rock fragmentation results at *D* of 3.8 and 4.3 mm, respectively, are similar, indicating that the macro cracks could no longer propagate once the indentation entered the unloading stage. This is because the dense core collapsed during the unloading stage, resulting in stress release and rapid reduction of the indentation force. This is further evidenced by the distribution of the force chains, in which two inverted triangles appear beneath the cutter tips inside of which the force chains are almost zero at the *D*_C_. Macro cracks generally developed along the indentation direction, and the propagation of side cracks was inadequate. This is because the studied rock was a hard granite (UCS ≈ 180 MPa), and it was not confined. This agrees with the results of previous studies [1,15,27] that the rock failure process changes from the formation of rock chips to a vertical axial splitting failure with decreasing confining pressure. The cutter indentation specific energy (*SE*) calculated by dividing the work of the cutter indentation force by the total fragment area was used to evaluate the rock fragmentation efficiency [14]. The average *SE* of the two CCS-type cutters was calculated as 4.8 MJ m^−3^.

As shown in figure 3*c,d*, the *F*_N_ of V-type cutter 1 and 2 increased linearly to their peak values of 1.00 × 10^7^ and 1.14 × 10^7^ N m^−1^, respectively, at *D* of 2.0 and 2.3 mm, respectively, and *D*_C_ is 2.7 mm. The dense cores were small. The main cracks were median cracks along the indentation direction, and the propagation of the side cracks was inadequate. Thus, no large chips were produced between the two V-type cutters. The average indentation *SE* of the two V-type cutters was calculated as 22.5 MJ m^−3^. Compared with that of the CCS model, the rock fragmentation of the V model was less efficient, even though the peak *F*_N_ and *D*_C_ of the V-type cutters were much smaller. This is because the rock fragments produced by the V-type cutters were fewer than those generated with the CCS-type cutters. In addition, the median crack length of the V model was shorter than that of the CCS model. These differences may be because the dense cores collapse more easily under the wedge indenter than under the flat intender.

## 4. Evolution of the mesoscopic indicators during the cutter indentation process

### 4.1. Coordination number

The coordination number (*Z*) represents the average number of particles in contact with each particle. This is an important factor used to evaluate the contact status, stress state and compactness of the particle system. *Z* can be calculated as follows [42]:

4.1 $$Z=\frac{2N_{C}}{N_{P}},$$

where *N*_C_ and *N*_P_ are the number of actual ‘ball–ball’ contacts and the total number of particles, respectively.

**Figure 4.**
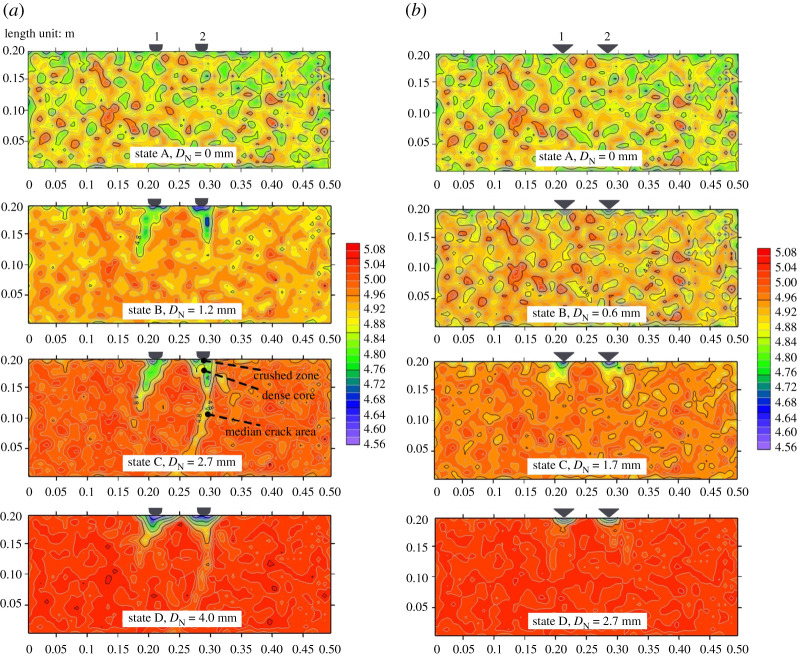
Distribution of the coordination number (*Z*) of the whole rock sample during the cutter indentation process: (*a*) CCS model; (*b*) V model.

To illustrate the evolution of the coordination number of the rock sample during the cutter indentation process, four states were selected for both the CCS model and the V model. According to figure 3, in state ‘A’, the initial state, the cutter has not penetrated the rock. State ‘B’ represents the early loading stage where the net indentation depth (*D*_N_) is small, state ‘C’ represents the later loading stage where the indentation force (*F*_N_) almost reaches its peak value, and state ‘D’ is the final state, where *D*_N_ equals to *D*_C_, and *F*_N_ drops to 0. The distribution of the contours of *Z* is shown in figure 4.

The distribution of *Z* in the initial state ‘A’ changed in different areas of the rock sample. This was because the rock was synthesized by randomly distributed particles whose radii varied between 0.4 and 0.6 mm, which represents the anisotropy and heterogeneity of the rock constituents at the microscopic scale. As the cutter penetrated the rock, the *Z* of the rock area away from the cutters increased gradually, and the distribution became uniform. For the CCS model in state ‘B’, the *Z* in the two elongated areas beneath the cutter was lower than that in the other areas. In addition, the *Z* of the two thin areas immediately beneath the cutter tips was especially low. This indicates that the thin rock areas were crushed, and slight dislocations occurred in the particles in the elongated areas. Then, when the cutter *D*_N_ reached 2.7 mm in state ‘C’, the *Z* of the thin areas became a little higher than that in state ‘B’, indicating the re-compaction of the crushed grains. In addition, the lengths of the elongated areas with low *Z* values were longer than those in state ‘B’, which is in accordance with the propagation of median cracks shown in figure 3. Finally, the model was in state ‘D’, where *D*_N_ is 4.0 mm. In this state, the *Z* of the rock areas beneath the cutter tip was the lowest compared with other states. The areas with low *Z* values coincided with the two inverted triangles with small contact force chains, as shown in figure 3*b*. Moreover, the *Z* value of the median crack areas became larger in state ‘D’ than that in state ‘C’. This illustrates the closure of the median cracks due to the rebound deformation of the bottom rock area after the collapse of the rock areas beneath the cutter tip. For the V model, a similar progressive evolution of *Z* could be observed during the cutter indentation. However, the fragmented areas were smaller, and the lengths of the median crack areas were shorter than those in the CCS model.

**Figure 5.**
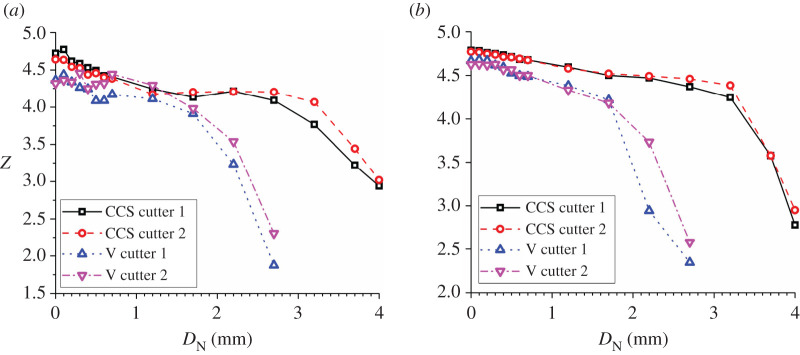
Evolution of the coordination number (*Z*) in the crushed zones (*a*) and the dense cores (*b*) during the cutter indentation process.

Accordingly, the rock beneath the cutter tip is divided into three areas, namely, the crushed zone, dense core and median crack area, as illustrated in figure 4*a*. The crushed zone is the thin area immediately beneath the cutter tip, where the grains are crushed and then re-compacted; the dense core represents the triangular area that bears the indentation force in the loading stage and then collapses in the unloading stage, and the median crack area represents the elongated area along the median crack that initiates from the bottom of the dense core. For further detailed analyses, the *Z* values of the crushed zones and dense cores of the CCS and V models were calculated, as shown in figure 5. The crushed zones of the CCS model are defined as two rectangles with widths and heights of 20 and 10 mm, respectively.

For the V model, the width and height of the crushed zone rectangles were 12 and 4 mm, respectively. The dense cores of the CCS model are defined as two equilateral triangles with a bottom and height of 70 and 30 mm, respectively. For the V model, the bottom and height of the dense core triangles were 40 and 14 mm, respectively. As shown in figure 5, the variations in the *Z* values for the two crushed zones or dense cores beneath the same type of cutters are similar to each other, indicating a similar rock indentation performance of the neighbouring cutters. For both the crushed zones and the dense cores, the *Z* of the CCS model was slightly larger than that of the V model initially when *D*_N_ was 0. This is caused by the difference in the sizes of the crushed zones and dense cores for the CCS and V models. As shown in figure 5*a*, the *Z* values of the crushed zones of the CCS model first decrease linearly, then tend to increase when *D*_N_ is larger than 1.7 mm, and finally decreases when *D*_N_ is larger than 3.2 mm. This variation in *Z* intuitively confirms the crushing and re-compaction of the grains in the crushed zone, which have been mentioned based on the distribution contours of *Z* in figure 4. Similar crushing and re-compaction phenomena can be found for the V model, but come earlier than the CCS model. As shown in figure 5*b*, the *Z* of the dense cores of the CCS model first decreases slowly and then decreases rapidly when the *D*_N_ is larger than 3.2 mm. This variation in *Z* intuitively represents the gradual crushing and sudden collapse of the dense core during the loading and unloading stages, respectively. A similar variation can be found for the V model, but the sudden drop occurs earlier when the *D*_N_ is approximately 1.8 mm, indicating that the dense core collapses more easily when indented by a V-type cutter than a CCS-type cutter. Comparing figure 5*a*,*b*, it can be summarized that (i) the re-compaction phenomenon only occurs in the thin crushed zone and (ii) the *Z* of the dense core is larger than that of the crushed zone.

### 4.2. Evolution of the meso-loop distribution

**Figure 6.**
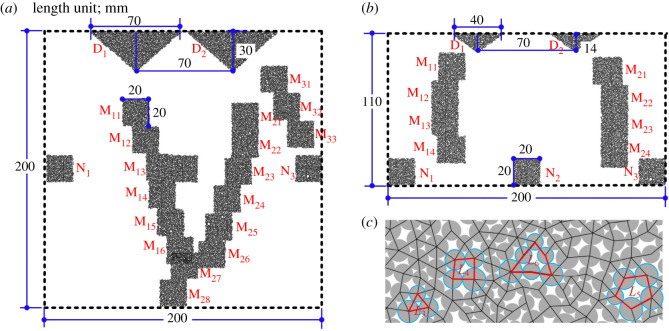
Extracted domains to investigate contact topological structure (meso-loop): (*a*) CCS model; (*b*) V model; (*c*) illustration of loops tessellated by contact branches.

Meso-loops are basic structures that conduct the mechanical behaviour of granular materials at the meso-scale. As shown in the figure 6*c*, the loops are defined as enclosed polygons by contact branches; hence, the evolution of the contact topological structure can be described by the geometrical evolution of the loops [36]. Meso-loops are categorized into eight orders according to their side number *i*, and are labelled as *L_i_* (*i* = 3, 4, 5, 6, 7, 8, 9, 10+), where ‘10+’ means a side number greater than or equal to 10. Based on the theorem that the sum of the edge vectors of a polygon is zero if they are ordered end-to-end, a program is proposed to obtain detailed information of all the loops, including the ID, coordinate and diameter of each particle located on the vertices of each loop, as well as the ID, vector and node information of each edge of each loop. For the studied numerical models that comprise approximately 113 000 particles and 276 000 contacts, it is very time consuming to obtain the detailed information of all the loops. Thus, the loops in some representative domains (i.e. the ‘dense core’ domain, the ‘median crack’ domain and the ‘none crack’ domain) were extracted and analysed. For the CCS model, as shown in figure 6*a*, a total of 22 domains were considered, including two ‘dense core’ domains (labelled as D_1_ and D_2_), 17 ‘median crack’ domains (crack 1 domains labelled as M_11_–M_16_, crack 2 domains labelled as M_21_–M_28_ and crack 3 domains labelled as M_31_–M_33_) and three ‘none crack’ domains (labelled as N_1_–N_3_). For the V model, as shown in figure 6*b*, a total of 13 domains were considered, including two ‘dense core’ domains (labelled as D_1_ and D_2_), eight ‘median crack’ domains (crack 1 domains labelled as M_11_–M_14_, and crack 2 domains labelled as M_21_–M_24_), and three ‘none crack’ domains (labelled as N_1_–N_3_).

**Figure 7.**
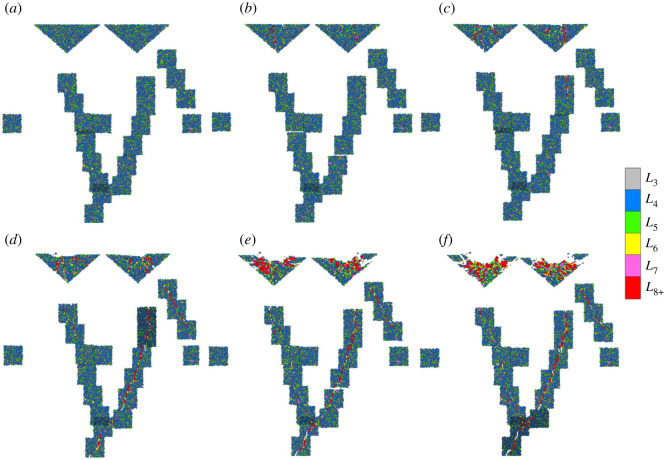
Distribution of contact topological structure (meso-loop) during cutter indentation process of the CCS model: (*a*) *D*_N_ = 0 mm; (*b*) *D*_N_ = 0.5 mm; (*c*) *D*_N_ = 1.2 mm; (*d*) *D*_N_ = 2.7 mm; (*e*) *D*_N_ = 3.7 mm; (*f*) *D*_N_ = 4.0 mm.

**Figure 8.**
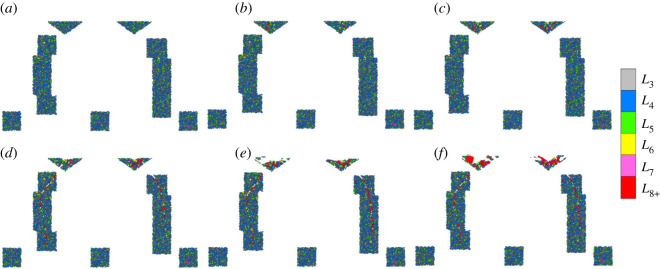
Distribution of contact topological structure (meso-loop) during cutter indentation process of the V model: (*a*) *D*_N_ = 0 mm; (*b*) *D*_N_ = 0.6 mm; (*c*) *D*_N_ = 1.2 mm; (*d*) *D*_N_ = 1.7 mm; (*e*) *D*_N_ = 2.2 mm; (*f*) *D*_N_ = 2.7 mm.

The initial meso-loops were mainly of a low order (*L*_3_ and *L*_4_), and only a few were of the order of *L*_5_. As the cutter penetrated the rocks, higher-order meso-loops formed gradually in the ‘dense core’ domains and the ‘median crack’ domains. During the loading stage, as illustrated in figure 7*b–d* and figure 8*b–d*, the positions of the high-order loops in the dense cores were basically in coincidence with the macro cracks that distribute radially, and the shapes of the high-order loops were more elongated than those of the low-order loops. This indicates the separation of the dense core into several large fragments along the radial cracks, and results in a gradual reduction in the bearing ability of the dense core. During the unloading stage (figure 7*e,f* and figure 8*e,f*), the number of high-order loops increased significantly in the dense cores, especially in the area directly beneath the cutter tip. This indicates that crushing and collapse of the dense core and rearrangement of the contact structure occurred. Therefore, as the internal solid fraction of the meso-loop tends to decrease, and more freedom is introduced into the loop structure to make it more deformable [35], as the loop order increases, the evolution of the bearing ability of the dense core is attributed to the order increment of the contact topological structure at the mesoscopic scale.

### 4.3. Evolution of the number proportions of meso-loops of different orders

**Figure 9.**
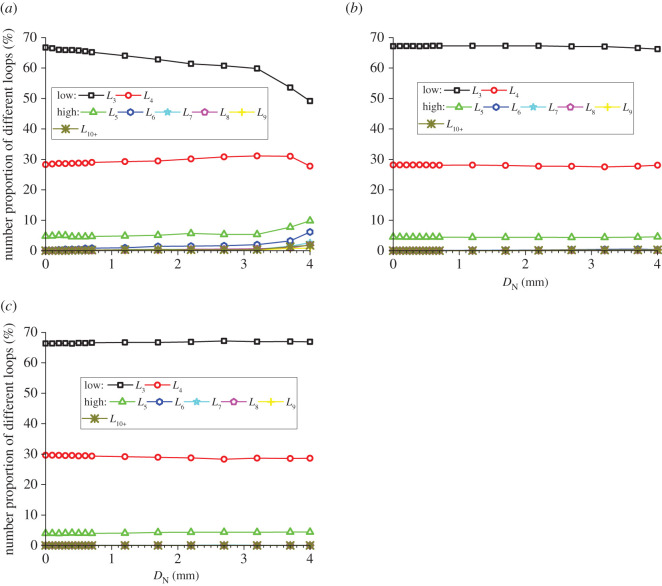
Evolution of the number proportions of different meso-loops during cutter indentation process of the CCS model: (*a*) the ‘dense core’ domains; (*b*) the ‘median crack’; (*c*) the ‘none crack’ domains.

The evolution of the number proportions of meso-loops of different orders is the most intuitive quantitative reflection of the evolution of the meso-structure [39]. The changing curves of the number proportions of the different-order meso-loops of the CCS model are shown in figure 9. The meso-loops were mostly of a low order initially when *D*_N_ was zero. The number proportions of the *L*_3_- and *L*_4_-order meso-loops were approximately 66–67% and 27–29%, respectively, for all the studied domains. As shown in figure 9*c*, for the ‘none crack’ domains, the number proportions of meso-loops of different orders were almost constant during the indentation process, indicating that the rock meso-structure did not change. As shown in figure 9*b*, for the ‘median crack’ domains, the number proportions of low-order meso-loops slightly decreased, while the number proportions of high-order meso-loops slightly increased. For example, the number proportions of the *L*_3_- and *L*_4_-order meso-loops decreased by 1.4% and 0.2%, respectively, and the number proportions of the *L*_6_- and *L*_7_-order meso-loops increased from 0% to 0.37% and 0.24%, respectively. This is because the evolution of the rock meso-structure only occurred in the small domains along the median cracks. As shown in figure 9*a*, for the ‘dense core’ domains, the number proportions of meso-loops of different orders change significantly. The number proportions of different-order meso-loops in the initial stage and at the ends of the loading and unloading stages are listed in table 4, as are the calculated increments. During the loading stage, the proportion of the *L*_3_-order meso-loop decreased from 66.8% to 59.9%, the number proportion of the *L*_4_-order meso-loop increased from 28.3% to 31.1%, the number proportion of the *L*_5_-order meso-loop increased from 4.8% to 5.4%, and the number proportions of the *L*_6_-, *L*_7_-, *L*_8_-, *L*_9_- and *L*_10+_-order meso-loops increased from 0 or approximately 0 to 2.0%, 0.4%, 0.6%, 0.2% and 0.4%, respectively. This illustrates that only the most stable *L*_3_-order meso-loop decreased, while meso-loops of other orders increased during the loading stage. Meanwhile, the evolution of the number proportions of meso-loops in different orders was gradual and slow during the loading stage. By contrast, the evolution accelerated once the indentation entered the unloading stage, and obvious changes in the number proportions occurred in the short unloading stage, especially for the high-order meso-loops. For the *L*_4_-order meso-loop, the number proportion slightly decreased back to approximately the initial value during the unloading stage. This phenomenon reflects the destruction and recombination of mesostructures during the indentation process.

**Table 4.** Number proportions and increment of different-order loops in the ‘dense core’ domains in different indentation stages (‘L_end’ and ‘U_end’ mean the end of loading and unloading stage respectively, and ‘INCR (%)’ means the increment of the number proportion compared with the initial stage).

order	CCS model					V model				
	initial	L_end	INCR (%)	U_end	INCR (%)	initial	L_end	INCR (%)	U_end	INCR (%)
*L*_3_	66.8	59.9	−10.3	49.1	−26.5	66.2	60.8	−8.2	53.2	−19.6
*L*_4_	28.3	31.1	9.9	27.8	−1.8	27.7	26.7	−3.6	25.0	−9.7
*L*_5_	4.8	5.4	12.5	9.8	104.2	6.1	7.0	14.8	9.1	49.2
*L*_6_	0.2	2.0	900	6.1	2950	0.0	2.7	/	2.9	/
*L*_7_	0.0	0.4	/	2.6	/	0.0	1.2	/	3.2	/
*L*_8_	0.0	0.6	/	1.9	/	0.0	1.0	/	1.6	/
*L*_9_	0.0	0.2	/	0.9	/	0.0	0.5	/	1.0	/
*L*_10+_	0.0	0.4	/	1.8	/	0.0	0.2	/	3.9	/

**Figure 10.**
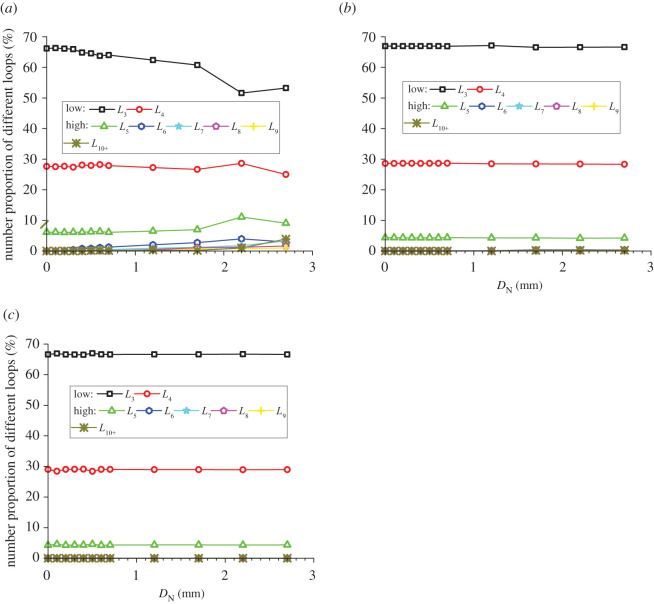
Evolution of the number proportions of different meso-loops during cutter indentation process of the V model: (*a*) the ‘dense core’ domains; (*b*) the ‘median crack’ domains; (*c*) the ‘none crack’ domains.

The changing curves of the proportions of the different-order meso-loops of the V model are shown in figure 10. The general change rules were similar to those of the CCS model, but the detailed change percentages were different. During the entire indentation process for the ‘dense core’ domains, the number proportions of the *L*_3_- and *L*_4_-order meso-loop decreased by 19.6% and 9.7%, respectively, while the proportion of the *L*_5_-order meso-loop increased by 49.2%.

The results of the above analyses reveal that the evolution of the meso-structures of the rock sample during indentation mainly occur in the ‘dense core’ domains; hence, further investigation of the meso-loop evolution will be conducted by mainly considering these domains. For the CCS and V models, the proportion of low-order meso-loops (*L*_3_ and *L*_4_) decreased from 100% to 76.9% and 78.2%, respectively, during the entire indentation process, indicating that the general evolution of the meso-structures was the same when the rock was indented by CCS- or V-type disc cutters. The major evolutions of the number proportions were a decrease in the *L*_3_- and *L*_4_-order meso-loops and an increase in the *L*_5_- and *L*_6_-order meso-loops, which mainly occurred in the unloading stage.

### 4.4. Evolution of the area percentages and average areas of meso-loops of different orders

**Figure 11.**
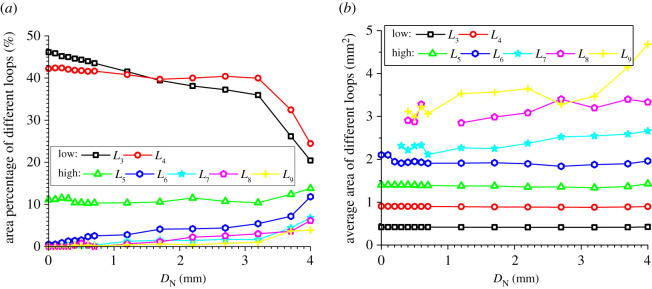
Evolution of the area percentage (*a*) and average area (*b*) of different meso-loops in the ‘dense core’ domains during cutter indentation process of the CCS model.

**Figure 12.**
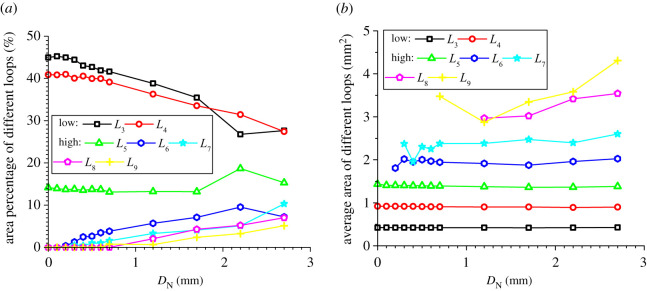
Evolution of the area percentage (*a*) and average area (*b*) of different meso-loops in the ‘dense core’ domains during cutter indentation process of the V model.

In addition to the number proportions of meso-loops of different orders, the evolution of the area percentages and average areas of meso-loops of different orders are also needed in order to represent the evolution of the meso-structure. The evolutions of the area percentage of meso-loops in the ‘dense core’ domains for the CCS and V models are illustrated in figures 11*a* and 12*a*, the evolution of the average area of the meso-loops are illustrated in figures 11*b* and 12*b*, and the area percentages and increments of different meso-loops in the ‘dense core’ domains in different indentation stages are shown in table 5. The *L*_10+_-order meso-loops were not analysed because the variability of the area of the *L*_10+_-order meso-loops was too high. For the CCS model, the general change rules of the area percentages were similar to those of the number proportions of different-order meso-loops. The initial area percentages of the *L*_3_- and *L*_4_-order meso-loops were approximately 46% and 42%, respectively. During the loading stage, the area percentages of the low-order meso-loops (*L*_3_ and *L*_4_) decreased steadily and slowly to a sum of 77.8%, while the area percentages of the high-order meso-loops (*L*_5_, *L*_6_, *L*_7_, *L*_8_ and *L*_9_) increased steadily and slowly to a sum of 22.2%.

**Table 5.** Area percentages and increment of different-order loops in the ‘dense core’ domains in different indentation stages (‘L_end’ and ‘U_end’ mean the end of loading and unloading stage respectively, and ‘INCR (%)’ means the increment of the number proportion compared with the initial stage).

order	CCS model					V model				
	initial	L_end	INCR (%)	U_end	INCR (%)	initial	L_end	INCR (%)	U_end	INCR (%)
*L*_3_	46.1	36.8	−20.0	23.4	−49.3	45.0	35.5	−21.1	27.6	−38.5
*L*_4_	42.2	41.0	−2.9	28.0	−33.8	40.9	33.5	−18.0	27.4	−32.9
*L*_5_	11.1	10.7	−3.7	15.8	42.4	14.2	13.2	−6.9	15.3	8.2
*L*_6_	0.5	5.6	919	13.5	2373	0.0	7.1	/	7.2	/
*L*_7_	0.0	1.7	/	7.7	/	0.0	4.1	/	10.3	/
*L*_8_	0.0	3.1	/	7.1	/	0.0	4.3	/	7.0	/
*L*_9_	0.0	1.1	/	4.5	/	0.0	2.4	/	5.1	/

By contrast, during the unloading stage, the area percentages of the low-order meso-loops (*L*_3_ and *L*_4_) decreased significantly to a sum of 51.4%, while the area percentages of the high-order meso-loops (*L*_5_, *L*_6_, *L*_7_, *L*_8_ and *L*_9_) increased significantly to a sum of 48.6%. As shown in figure 11*b*, the average areas of the low-order meso-loops are generally constant during the indentation process, while the average areas of the high-order meso-loops (especially *L*_9_) improve as the cutters penetrate the rock, and the higher the meso-loop order, the more sensitive the average area to the indentation depth. In addition, as discussed in §4.3, the number proportions of the low- and high-order meso-loops decreased and increased, respectively, during the indentation process. These are the reasons for the decrease and increase in the area percentages of the low- and high-order meso-loops, respectively. As the average meso-loop area is a rough representation of the material porosity, the decrease in the number proportions and area percentages of low-order meso-loops and the increment of the number proportions and area percentages of high-order meso-loops result in the volume expansion and collapse of the dense core. Hence, the volume expansion of the dense core was not significant during the loading stage. In addition, it has been discussed in §3 that the propagation of macroscopic cracks mainly occurs in the loading stage. As a result, it may be inferred that the expansion of the dense core is not the principal cause of macroscopic crack propagation. For the V model, the breakover of the area percentage curves at the end of the loading stage was not as remarkable as that for the CCS model. During the entire indentation process, the area percentages of the low-order (*L*_3_ and *L*_4_) meso-loops continued to decrease from a sum of 100% to 55.0%.

### 4.5. Evolution of the internal pore area and porosity of meso-loops of different orders

**Figure 13.**
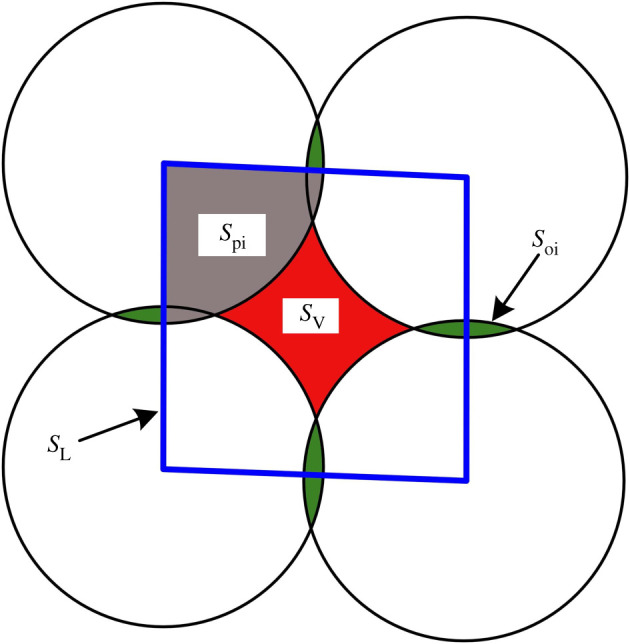
Sketch of the calculation of the internal pore area of a meso-loop.

As shown in figure 13, the area of a meso-loop (*S*_L_) is composed of two parts: the covered particle area (*S*_pi_) and the internal pore area (*S*_V_). As the particles are usually assumed to be undeformable, it may be more rational and accurate to attribute the volume change of the ‘dense core’ domains to the evolution of the internal pore area of the meso-loops. According to Ling *et al*. [33], the internal pore area of a meso-loop was calculated using equation (4.2).

4.2 $$S_{V}=S_{L}-\sum S_{\mathrm{pi}}+0.5\sum S_{\mathrm{oi}},$$

where *S*_V_ denotes the area of the internal pore; *S*_L_ denotes the area of a meso-loop that has been calculated in §4.4, *S*_pi_ denotes the area of a particle region that is covered by the meso-loop and *S*_oi_ denotes the overlapping area of neighbouring particles.

Hence, the porosity of a meso-loop (PL) was calculated using equation (4.3).

4.3 $$P_{L}=\frac{S_{V}}{S_{L}}.$$

**Figure 14.**
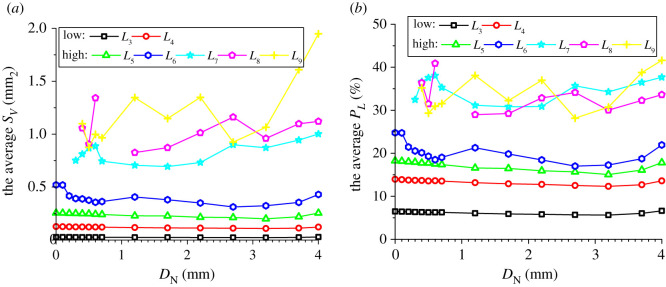
The average internal pore area (*S*_V_) (*a*) and porosity (*P*_L_) (*b*) of meso-loops in the ‘dense core’ domains during cutter indentation process of the CCS model.

As noted in §4.4, the internal pores of the *L*_3_- to *L*_9_-order meso-loops were studied, while the *L*_10_- order meso-loops were not considered. For the CCS model, the evolution of the average *S*_V_ and *P*_L_ of meso-loops of different orders in the ‘dense core’ domains during indentation are shown in figure 14. The results revealed that the change rules of the average *S*_V_ were similar to those of the average loop area illustrated in figure 11*b*, as the particles were undeformable. First, the average *S*_V_ increased with increasing loop order, which agrees with the fact that the internal solid fraction of the meso-loop tends to decrease as the loop order increases. Furthermore, the average *S*_V_ of the *L*_3_-, *L*_4_- and *L*_5_-order meso-loops were constant during cutter indentation, while the average *S*_V_ of the high-order meso-loops (especially *L*_9_) improved as the cutters penetrated, and the higher the meso-loop order, the larger the increment of the average *S*_V_ during the entire indentation process. This indicates that the volume expansion of the dense core is mainly caused by the increment in the internal pore area of the high-order meso-loops. The average *P*_L_ increased with an increase in the loop order when the order was lower than *L*_6_, and the average *P*_L_ of the *L*_7_-, *L*_8_- and *L*_9_-order meso-loops were not significantly different. During the loading stage, the average *P*_L_ of different-order meso-loops generally decreased with an increase in *D*_N_; subsequently, the average *P*_L_ tended to increase with increasing *D*_N_ during the unloading stage. This change rule was especially evident for the *L*_6_- and higher-order loops. This indicates that the meso-loops were compressed during the loading stage and then expanded during the unloading stage.

**Figure 15.**
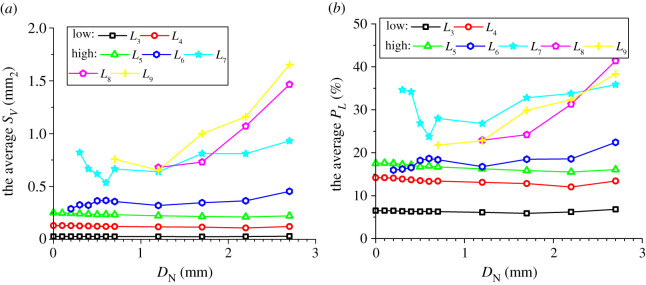
The average internal pore area (*S*_V_) (*a*) and porosity (*P*_L_) (*b*) of meso-loops in the ‘dense core’ domains during cutter indentation process of the V model.

In the case of the V model, as shown in figure 15, the change rules of the average *S*_V_ with *D*_N_ were similar to those of the average loop area illustrated in figure 12*b*. First, the average *S*_V_ increased with the loop order. Then, the average *S*_V_ of the *L*_3_- to *L*_6_-order meso-loops did not change with *D*_N_, while the average *S*_V_ of the *L*_6+_-order meso-loops increased significantly with an increase in *D*_N_. This means that the volume expansion of the dense core was mainly caused by the increment in the internal pore area of high-order meso-loops that had low internal solid fractions. The average *P*_L_ of the *L*_3_- to *L*_6_-order meso-loops increased with the loop order, and the average *P*_L_ of the *L*_7_-, *L*_8_- and *L*_9_-order meso-loops did not show a clear change rule with the loop order. The average *P*_L_ of the *L*_3_-, *L*_4_- and *L*_5_-order meso-loops was generally constant during the indentation process, while the average *P*_L_ of the *L*_6+_-order meso-loops generally increased with an increase in *D*_N_. This indicates that no obvious volume compression of the meso-loops occurred during the loading stage when the rock was indented by a V cutter.

### 4.6. Evolution of the shape of meso-loops of different orders

**Figure 16.**
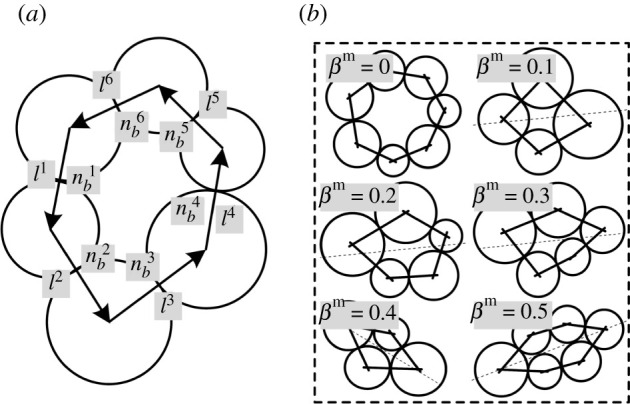
Illustration of tensor components of a meso-loop (*a*) and meso-loops with different elongation degrees (*b*) (after Cambou *et al.* [43]).

The shape of a meso-loop was evaluated using the elongation degree (*β*^m^) calculated from the tensor (*L*^m^) of the meso-loop [43]. The loop tensor *L*^m^ is represented by formulae (4.4) and (4.5), and its trace measures the meso-loop perimeter, and the elongation degree (*β*^m^) is calculated by formula (4.6). The components, *l^k^* and *n_b_^k^*, are shown in figure 16*a*.

4.4 $$L^{m}=\sum_{k=1}^{r^{m}}l^{k}n_{b}^{k}⊗n_{b}^{k},$$

4.5 $$L^{m}=\frac{1}{2}\mathrm{Trace}(L^{m})I+D^{m},$$

and

4.6 $$\;\beta^{m}=\frac{\left\|D^{m}\right\|}{\mathrm{Trace}(L^{m})}=\sqrt{\frac{1}{2}}\frac{L_{1}^{m}-L_{2}^{m}}{L_{1}^{m}+L_{2}^{m}},$$

where ***l****^k^* is the branch vector at contact *k*; $l^{k}=l^{k}n_{b}^{k}$, with length *l^k^* and unit vector $n_{b}^{k}$ directed along branch *k*; *r*^m^ is the number of sides of meso-loop *m*; and ***D***^m^ is the deviatoric part of loop tensor ***L***^m^ which characterizes the shape of the meso-loop under consideration. $L_{1}^{m}$ and $L_{2}^{m}$ are the major and minor eigenvalues of ***L***^m^, respectively.

**Figure 17.**
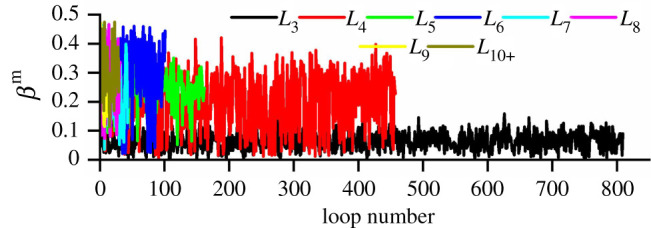
The elongation degree, *β*^m^, of meso-loops with different orders in the ‘dense core’ domains of the CCS model when *D*_N_ is 4.0 mm.

As shown in figure 16*b*, the elongation degree, *β*^m^, was zero for a meso-loop of regular shape, and the higher the elongation degree, *β*^m^, the greater the elongation of the meso-loop [43]. The *β*^m^ of the meso-loops in the ‘dense core’ domains of the CCS model when *D*_N_ was 4.0 mm are shown in figure 17. It can be seen that the number of meso-loops decreased with increasing meso-loop order, which has been discussed in detail in §4.3. Furthermore, *β*^m^ generally increased with increasing loop order, indicating that the higher the loop order, the more elongated the meso-loop. This is because the high-order meso-loops mainly occur along macroscopic cracks. Moreover, it was found that the fluctuation amplitude of the *L*_3_-order meso-loop was much smaller than that of the other meso-loops, as the *L*_3_-order meso-loop was the most stable and usually had a regular shape.

**Figure 18.**
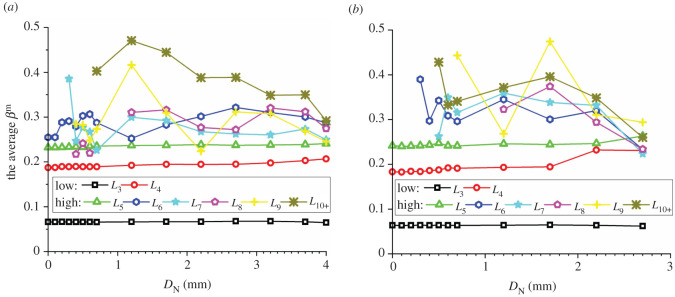
The average *β*^m^ of meso-loops with different orders in the ‘dense core’ domains for the CCS model (*a*), and the V model (*b*).

To study the evolution of the shapes of different-order meso-loops during cutter indentation, the average values of *β*^m^ of different-order meso-loops in the ‘dense core’ domains for the CCS and V models were calculated and are shown in figure 18. The results confirm the above finding that *β*^m^ generally increases with increasing loop order. The average *β*^m^ of the *L*_3_-order meso-loop was much smaller than those of the other meso-loops, and it was constant during the indentation process. The average *β*^m^ of the *L*_4_- and *L*_5_-order meso-loops were also basically constant during cutter indentation, but they were evidently larger than that of the *L*_3_-order meso-loop. As meso-loops are the basic structure responsible for the mechanical behaviour of granular material, the constant shape and dominant proportion of low-order meso-loops during indentation indicate that the *L*_3_-, *L*_4_- and *L*_5_-order meso-loops are the principal meso-loops that bear and transfer the indentation force.

For the CCS model, the average *β*^m^ of the L_6_-order meso-loops increased from the initial value of 0.25 to 0.31 when *D*_N_ was 0.6 mm. Subsequently, it continued to decrease to 0.25 as the cutters continued to penetrate the rock and as *D*_N_ increased to 1.2 mm; then, it tended to increase to approximately 0.32 during the following loading stages. As the *L*_6_-order meso-loop is a link between the low- and high-order meso-loops, the evolution of its shape is discussed to explain the initiation and propagation of cracks in the ‘dense core’ domains at the mesoscopic scale. In general, the entire crack evolution process in the ‘dense core’ domains can be divided into three stages based on the indentation depths of 0–0.6, 0.6–1.2 and 1.2–3.0 mm, respectively, according to the change rule of the average *β*^m^ of the *L*_6_-order meso-loops.

**Figure 19.**
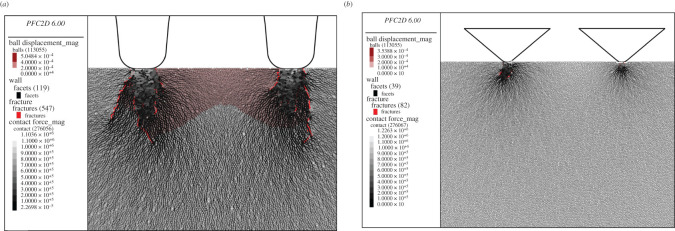
Distribution of the ball displacement, contact force and fractures beneath the cutter tip when *D*_N_ is 0.5 mm for the CCS model (*a*), and *D*_N_ is 0.6 mm for the V model (*b*). ©2020 Itasca consulting group, Inc.

As shown in figure 19, during the first stage of local crack initiation, two dense and strong contact force regions with an outline similar to the Hertzian stress ellipse are formed directly beneath the cutter tips. Because of the displacement inconformity of the particles in the boundary of the strong contact force regions, the bonds between neighbouring particles are broken and illustrated in ‘fractures’. The connections and constraints are thus reduced, and hence dislocation and shear dilation occur as the particles are driven to move by the contact force chains, causing the development of *L*_3_-, *L*_4_- and *L*_5_-order meso-loops to *L*_6_- and higher-order meso-loops (see figure 7*b*). As the newborn *L*_6_-order meso-loop is usually narrow and elongated in shape, the average *β*^m^ increases accordingly. It has been noted previously that *L*_6_- and higher-order meso-loops are more deformable than the *L*_3_-, *L*_4_- and *L*_5_-order meso-loops as more particle freedom is introduced. Therefore, the scattered distributed high-order meso-loops can be regarded as the initiation of local cracks in the ‘dense core’ domains.

The second stage mainly includes the processes of local crack mergence and macro crack formation. As the cutters penetrates the rock, the elongated *L*_6_-order meso-loops are compressed to expand laterally and hence become more regular. Meanwhile, the low-order meso-loops around the local cracks are forced to develop into high-order meso-loops, causing the merging of neighbouring local cracks and the formation of macro cracks, as illustrated in figure 7*c*. The third stage mainly includes the processes of macro crack propagation and local crack generation. As the macro cracks propagate, the ‘dense core’ domains are broken into several fragments (see figure 7*d*) and the force bearing ability reduces gradually, causing the generation of more elongated *L*_6_- and higher-order meso-loops from low-order meso-loops, which can explain the increase in the average *β*^m^. For the *L*_7+_-order meso-loops, the change rule of the average *β*^m^ with *D*_N_ was not evident, as there were only a few high-order meso-loops, and their shapes varied significantly during cutter indentation.

For the V model, the average *β*^m^ of the *L*_3_-, *L*_4_- and *L*_5_-order meso-loops were generally constant during the indentation process, and the average *β*^m^ of the *L*_6_ and higher-order meso-loops were higher than those of the low-order meso-loops; these results are similar to those of the CCS model. The variation in the average *β*^m^ of the *L*_6_-order meso-loops with the *D*_N_ of the V model was similar to that of the CCS model. The main difference existed at the beginning, as there were no initial *L*_6_-order meso-loops in the studied ‘dense core’ domains for the V model. The subsequent decrement and then the increment of the average *β*^m^ of the *L*_6_- and higher-order meso-loops can be explained by the following assumptions: (i) local crack mergence and macro crack formation, and (ii) macro crack propagation and more local crack generation. The contact force regions and the initiation of the ‘fractures’ in the V model are illustrated in figure 19 when *D*_N_ is 0.6 mm. As the contact area between the sharp V-type cutter and rock was very small, the contact force regions were small, and particle displacement mainly occurred. Even so, it confirms the conclusion drawn from the CCS model analysis that the initial ‘fractures’ come into being due to the displacement inconformity of the particles in the contact force region boundary.

For both the CCS and V models, the average *β*^m^ of the *L*_7+_-order meso-loops decreased to low values once the indentation entered the unloading stage. This indicates that the shape of the *L*_7+_-order meso-loops becomes regular during the collapse of the ‘dense core’ domains, as the meso-loops evolve freely without the strong contact force chains, as shown in figure 3*b,d*. This result is in accordance with the results discussed in §§4.4 and 4.5 on the evolution of the average area of the meso-loop and the internal pore. For a more detailed analysis, the meso-loop can be simplified as an ellipse that is fitted by the vertices of the loop, according to Liu *et al.* [37–39]. The ratio *i* of the major axis length to the minor axis length can be regarded as an indicator of the meso-loop shape, and *i* is always not less than 1. The larger the value of i, the more elongated the meso-loop, which is similar to the trend for indicator *β*^m^. The area of an ellipse *SE* can be calculated using equation (4.7) once the ellipse perimeter *L* and ratio *i* are known. For a certain meso-loop simplified by an ellipse, the perimeter *L* is essentially constant during the shape evolution. Thus, it is easy to determine that the *SE* decreases with increasing *i*. Therefore, the results that (i) the average area of high-order meso-loops increases with *D*_N_, as illustrated in figures 11*b* and 12*b*, and that (ii) the average *β*^m^ of the high-order meso-loops decreases with *D*_N_, as illustrated in figure 18, can confirm each other.

4.7 $$SE=\frac{L^{2}i}{{\left(4i+2\pi +4\right)}^{2}}.$$

Based on the above analyses, some additional summaries and assumptions are discussed. For simulations using the PFC software, the macroscopic crack is usually represented by the built-in ‘fracture’, which is inserted in the contact position once the contact bond is broken. However, it might be inappropriate for representing macro cracks in the ‘dense core’ domains during cutter indentation. This is because the breakage of a contact bond is not equivalent to the disappearance of the contact itself. For example, as shown in figure 7*b* and figure 17*a*, many ‘fractures’ come into being during the early loading stage; however, most particles are compacted, and few macro cracks appear owing to the constraint of the cutter tip. By contrast, the distribution contour of the meso-loops that are coloured according to the loop order may be more appropriate and intuitive for representing the evolution of cracks on a macroscopic scale. The initiation and propagation of cracks in the ‘dense core’ domains are attributed to the combination of external and internal causes. The main external causes are the contact force chains and the boundary conditions, while the main internal cause is the evolution of mesoscopic contact structures, which are also called meso-loops. Therefore, the reason for crack growth on the mesoscopic scale is assumed to be the evolution of low-order meso-loops to high-order meso-loops driven by contact force chains.

### 4.7. Influence of particle packing parameters on mesoscopic structures

In the DEM, the mesoscopic mechanical structures, especially the coordination number and meso-loops, are strongly influenced by the factors of particle packing, including particle size or grain size, particle size distribution, porosity, etc. According to previous publications [22,31,32,40], there are some commonly used ranges or assumptions for the packing parameters of rock material aggregated by particles: (i) the particle radius is usually 0.1–0.9 mm considering the model accuracy and simulation cost, (ii) the ratio of maximum particle radius to minimum particle radius is usually 1.5–2.0, (iii) the porosity of particle-synthesized rock is usually 0.1–0.15 for two-dimensional models, and (ivd) the grain size is usually adjusted by the quantity of grains according to laboratory scan. Based on these assumptions, another two sets of particle packing parameters were selected different from those of the existing model (sample-0) to synthesize two new rock samples (sample-1 and -2), and the particle contact parameters were re-calibrated and were different from those of sample-0, as shown in tables 2 and 3. It indicates that the macroscopic mechanical properties of the rock sample are sensitive to particle packing and contact parameters.

**Figure 20.**
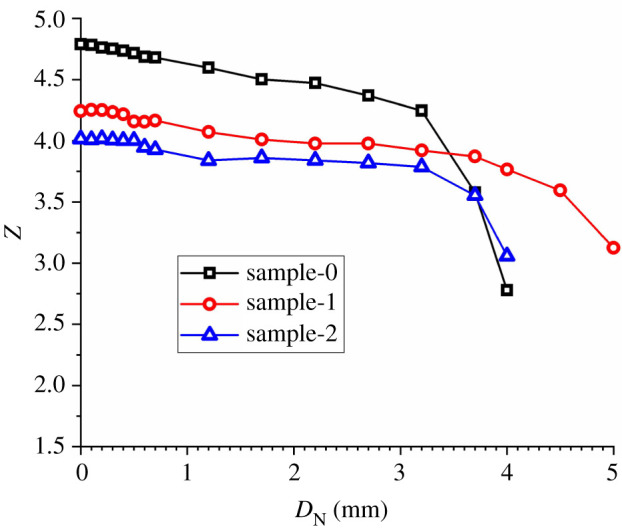
Evolution of the coordination number (*Z*) in the dense core zones of cutter 1 during the cutter indentation process conducted on different rock samples.

**Figure 21.**
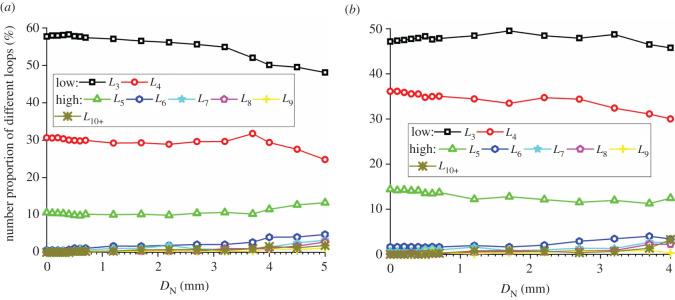
Evolution of the number proportions of different meso-loops during cutter indentation process of the CCS model in the ‘dense core’ domains: (*a*) sample-1; (*b*) sample-2.

To study the influence of particle packing parameters on mesoscopic structures, another two simulations of the rock indentation process by CCS cutters were conducted. For the ‘dense core’ domains of the three simulations, the coordination number (*Z*) and number proportions of the meso-loops with different orders were selected as representative mesoscopic structure indicators and were analysed. As shown in figure 20, the initial *Z* values of the three models are different, indicating that the coordination number is sensitive to the particle packing parameters. However, the general trends of the evolution of the *Z* value for the three models are like each other. This variation in *Z* intuitively represents the gradual crushing and sudden collapse of the dense core during the loading and unloading stages, respectively. As shown in figure 21 and figure 9*a*, the initial number proportions of the meso-loops with different orders for the three simulations are different, indicating that the meso-loop distribution is sensitive to the particle packing parameters. However, the *L*_3_-, *L*_4_- and *L*_5_-order meso-loops account for the most proportion for the three simulations. Besides, the general trends of the evolution of the number proportions of different-order meso-loops are similar for the three simulations. It reflects the destruction and recombination of mesostructures during the indentation process in the ‘dense core’ domains.

According to the above analysis, it is found that the mesoscopic mechanical structures are sensitive to the particle packing parameters. However, the general evolution trends of the mesoscopic structures are like each other for the simulations conducted on the rock samples with different particle packing parameters. It indicates that the conclusions drawn in this study are universal to some extent.

## 5. Conclusion

This study mainly investigated the geometric evolution of mesoscopic mechanical structures in rock fragmentation processes induced by TBM cutters. The rock indentation models were built using a GB-DEM considering CCS- and V-type cutters. The evolution of the mesoscopic indicators, including the coordination number, meso-loop distribution, number, area, porosity and shape, were investigated, and the relationship between the mesoscopic evolution and the macroscopic response of rock is discussed. The following conclusions can be drawn:

(1) Macro cracks mainly grow in the loading stage as the collapse of the dense core in the unloading stage results in stress release and thus a rapid reduction in the indentation force. The rock-breaking efficiency evaluated by the indentation specific energy is much higher for the CCS model than for the V model.
(2) According to the evolution of the coordination number, crushing and re-compaction of the mineral grains mainly occur in the thin crushed zone immediately beneath the cutter tip, while the ‘dense core’ is gradually crushed and then suddenly collapses during the loading and unloading stages respectively.
(3) According to the evolution of the meso-loop distribution, it can be inferred that the reduction in the bearing ability of the dense core during cutter indentation is attributed to the order increment of the contact topological structure at the mesoscopic scale.
(4) The evolution of the meso-structures during indentation mainly occurs in the ‘dense core’ domains. The major evolutions of the number proportions are a decrease in *L*_3_- and *L*_4_-order meso-loops and an increase in *L*_5_- and *L*_6_-order meso-loops, which mainly occurs in the unloading stage.
(5) The area percentages of the low- and high-order meso-loops decrease and increase, respectively, during the indentation process. The expansion of the dense core is inferred not to be the principal cause of macroscopic crack propagation. The volume expansion of the dense core is mainly caused by the increase in the internal pore area of high-order meso-loops with low internal solid fractions.
(6) According to the shape analysis of the meso-loops, the higher the loop order, the greater the elongation of the meso-loop during cutter indentation. The constant shape and dominant proportion of the low-order meso-loops during cutter indentation indicate that the *L*_3_-, *L*_4_- and *L*_5_- order meso-loops are the principal meso-loops that bear and transfer the indentation force. The shape of the high-order meso-loops becomes regular as the cutter penetrates during the unloading stage.
(7) The distribution contour of the meso-loops that are coloured according to the loop order is suggested to be an appropriate and intuitive approach for representing the evolution of cracks on a macroscopic scale. The reason for crack growth on the mesoscopic scale is assumed to be the evolution of low-order meso-loops to high-order meso-loops driven by contact force chains.

The main focus of this study was to provide new insights into the development of a dense core during the cutter indentation process from the perspective of the geometric evolution of the mesoscopic mechanical structure of rock. Limited by the length of this article, we were not able to thoroughly investigate the influence of parameters such as the cutter spacing, joint structure and confining stress.

## Data accessibility

The data supporting this research are provided in the tables and figures embedded in the article. The modelling files were uploaded as electronic supplementary material, files. The nine electronic supplementary material, files are ‘0.prj’, ‘cutter1.dxf’, ‘cutter2.dxf’, ‘fracture.p2fis’, ‘make-sample.dat’, ‘make-cluster.dat’, ‘bonds.dat’, ‘penetrate.dat’ and ‘setup.p2fis’ [44].

## References

[1] Liu H, Kou S, Lindqvist P. 2002 Numerical simulation of the rock fragmentation process induced by indenters. Int. J. Rock Mech. Min. Sci. **39**, 491-505. (10.1016/S1365-1609(02)00043-6)

[2] Gong QM, Jiao YY, Zhao J. 2006 Numerical modelling of the effects of joint spacing on rock fragmentation by TBM cutters. Tunn. Undergr. Space Technol. **21**, 46-55. (10.1016/j.tust.2005.06.004)

[3] Gong QM, Zhao J, Jiao YY. 2005 Numerical modeling of the effects of joint orientation on rock fragmentation by TBM cutters. Tunn. Undergr. Space Technol. **20**, 183-191. (10.1016/j.tust.2004.08.006)

[4] Innaurato NOC et al. 2007 Experimental and numerical studies on rock breaking with TBM tools under high stress confinement. Rock Mech. Rock Eng. **40**, 429-451. (10.1007/s00603-006-0109-4)

[5] Su L, Sun J, Lu W. 2009 Research on numerical simulation of rock fragmentation by TBM cutters using particle flow method. Rock Soil Mech. **30**, 2823-2829. (10.1016/S1874-8651(10)60073-7)

[6] Tan Q, Zhang K, Zhou ZL, Xia YM. 2010 Numerical simulation and experimental observation of rock cracks under action of spherical tooth hob cutter. Chinese J. Rock Mech. Eng. **29**, 163-169.

[7] Cho JW, Jeon S. 2010 Optimum spacing of TBM disc cutters: a numerical simulation using the three-dimensional dynamic fracturing method. Tunn. Undergr. Space Technol. **25**, 230-244. (10.1016/j.tust.2009.11.007)

[8] Cho JW, Jeon S. 2013 Evaluation of cutting efficiency during TBM disc cutter excavation within a Korean granitic rock using linear-cutting-machine testing and photogrammetric measurement. Tunn. Undergr. Space Technol. **35**, 37-54. (10.1016/j.tust.2012.08.006)

[9] Sun JS, Chen M, Chen BG, Lu WB, Zhou CB. 2011 Numerical simulation of influence factors for rock fragmentation by TBM cutters. Rock Soil Mech. **32**, 1891-1897. (10.16285/j.rsm.2011.06.026)

[10] Ma H, Yin L, Ji H. 2011 Numerical study of the effect of confining stress on rock fragmentation by TBM cutters. Int. J. Rock Mech. Min. Sci. **48**, 1021-1033. (10.1016/j.ijrmms.2011.05.002)

[11] Mo ZZ, Li HB, Zhou QC, He EG, Zou F, Zhu XM, Zhao Y. 2012 Research on numerical simulation of rock breaking using TBM disc cutters based on UDEC method. Rock Soil Mech. **33**, 1196-1202. (10.16285/j.rsm.2012.04.033)

[12] Tan Q, Yi N, Xia Y, Xu Z, Zhu Y, Song J. 2012 Research on rock dynamic fragmentation characteristics by TBM cutters and cutter spacing optimization. Chinese J. Rock Mech. Eng. **31**, 2453-2464.

[13] Tan Q, Li J, Xia Y, Xu Z, Zhu Y, Zhang J. 2013 Numerical research on rock fragmentation process by disc cutter. Rock Soil Mech. **34**, 2707-2714. (10.16285/j.rsm.2013.09.042)

[14] Moon T, Oh J. 2012 A study of optimal rock-cutting conditions for hard rock TBM using the discrete element method. Rock Mech. Rock Eng. **45**, 837-849. (10.1007/s00603-011-0180-3)

[15] Peng Q. 2014 Research on influence mechanism of confining pressure on rock breakage by TBM cutters. Chinese J. Rock Mech. Eng. **33**, 2743-2749. (10.13722/j.cnki.jrme.2014.s1.022)

[16] Choi SO, Lee SJ. 2014 Three-dimensional numerical analysis of the rock-cutting behavior of a disc cutter using particle flow code. KSCE J. Civ. Eng. **19**, 1129-1138. (10.1007/s12205-013-0622-4)

[17] Liu J, Cao P, Jiang Z, Liu J, Tan X. 2015 Simulation of TBM cutter penetration under static and dynamic coupled loads. J. Cent. South Univ. **46**, 1393-1401.

[18] Han M, Cai Z, Qu C, Chen K. 2015 Tunneling simulation and strength analysis of cutterhead system of TBM. In 8th Int. Conf., ICIRA, pp. 445-455. Portsmouth, UK: Springer.

[19] Han MD, Cai ZX, Qu CY, Jin LS. 2017 Dynamic numerical simulation of cutterhead loads in TBM tunnelling. Tunn. Undergr. Space Technol. **70**, 286-298. (10.1016/j.tust.2017.08.028)

[20] Yang H, Wang H, Zhou X. 2015 Analysis on the rock-cutter interaction mechanism during the TBM tunneling process. Rock Mech. Rock Eng. **49**, 1073-1090. (10.1007/s00603-015-0796-9)

[21] Labra C, Rojek J, Oñate E. 2016 Discrete/finite element modelling of rock cutting with a TBM disc cutter. Rock Mech. Rock Eng. **50**, 621-638. (10.1007/s00603-016-1133-7)

[22] Li XF, Li HB, Liu YQ, Zhou QC, Xia X. 2016 Numerical simulation of rock fragmentation mechanisms subject to wedge penetration for TBMs. Tunn. Undergr. Space Technol. **53**, 96-108. (10.1016/j.tust.2015.12.010)

[23] Geng Q, Wei Z, Meng H, Chen Q. 2016 Numerical and experimental research on the rock-breaking process of tunnel boring machine normal disc cutters. J. Mech. Sci. Technol. **30**, 1733-1745. (10.1007/s12206-016-0329-9)

[24] Geng Q, Wei Z, Ren J. 2017 New rock material definition strategy for FEM simulation of the rock cutting process by TBM disc cutters. Tunn. Undergr. Space Technol. **65**, 179-186. (10.1016/j.tust.2017.03.001)

[25] Xia Y, Guo B, Cong G, Zhang X, Zeng G. 2017 Numerical simulation of rock fragmentation induced by a single TBM disc cutter close to a side free surface. Int. J. Rock Mech. Min. Sci. **91**, 40-48. (10.1016/j.ijrmms.2016.11.004)

[26] Xiao N, Zhou X, Gong Q. 2017 The modelling of rock breakage process by TBM rolling cutters using 3D FEM-SPH coupled method. Tunn. Undergr. Space Technol. **61**, 90-103. (10.1016/j.tust.2016.10.004)

[27] Liu Q, Jiang Y, Wu Z, Xu X, Liu Q. 2018 Investigation of the rock fragmentation process by a single TBM cutter using a Voronoi element-based numerical manifold method. Rock Mech. Rock Eng. **51**, 1137-1152. (10.1007/s00603-017-1381-1)

[28] Zhang XP, Ji PQ, Liu QS, Liu Q, Zhang Q, Peng ZH. 2018 Physical and numerical studies of rock fragmentation subject to wedge cutter indentation in the mixed ground. Tunn. Undergr. Space Technol. **71**, 354-365. (10.1016/j.tust.2017.09.003)

[29] Zhang Z, Zhang K, Dong W, Zhang B. 2020 Study of rock-cutting process by disc cutters in mixed ground based on three-dimensional particle flow model. Rock Mech. Rock Eng. **53**, 3485-3506. (10.1007/s00603-020-02118-y)

[30] Xu H, Geng Q, Sun Z, Qi Z. 2021 Full-scale granite cutting experiments using tunnel boring machine disc cutters at different free-face conditions. Tunn. Undergr. Space Technol. **108**, 103719. (10.1016/j.tust.2020.103719)

[31] Cho N, Martin CD, Sego DC. 2007 A clumped particle model for rock. Int. J. Rock Mech. Min. Sci. **44**, 997-1010. (10.1016/j.ijrmms.2007.02.002)

[32] Li XF, Li HB, Zhao J. 2017 3D polycrystalline discrete element method (3PDEM) for simulation of crack initiation and propagation in granular rock. Comput. Geotech. **90**, 96-112. (10.1016/j.compgeo.2017.05.023)

[33] Ling YY, Zhang Q, Wang XG, Zhao YF, Zhou JW. 2020 Discrete element analysis of deformation and failure characteristics of soil-rock mixture under flexible boundary biaxial compression. Rock Soil Mech. **41**, 1-11. (10.16285/j.rsm.2020.1285)

[34] Qin J, Chi L. 2013 Micromechanical analysis of dilatancy in granular materials. Rock Soil Mech. **34**, 1508-1514. (10.16285/j.rsm.2013.05.007)

[35] Zhu H, Nguyen HNG, Nicot F, Darve F. 2016 On a common critical state in localized and diffuse failure modes. J. Mech. Phys. Solids **95**, 112-131. (10.1016/j.jmps.2016.05.026)

[36] Zhu H, Nicot F, Darve F. 2016 Meso-structure organization in two-dimensional granular materials along biaxial loading path. Int. J. Solids Struct. **96**, 25-37. (10.1016/j.ijsolstr.2016.06.025)

[37] Liu Y, Wang CL, Zhang D. 2015 Distribution and evolution of pore structure in 2D granular materials under biaxial compression. Chinese J. Geotech. Eng. **37**, 494-503. (10.11779/CJGE201503013)

[38] Liu Y, Li S. 2018 Numerical simulation and analysis of meso-mechanical structure characteristic at critical state for granular media. Rock Soil Mech. **39**, 2237-2248. (10.16285/j.rsm.2016.1966)

[39] Liu Y, Li S, Wu KJ. 2018 Geometry evolution and stability analysis for mesoscopic mechanical structure in shear band of granular media. Chinese J. Rock Mech. Eng. **37**, 3686-3700. (10.13722/j.cnki.jrme.2016.1087)

[40] Potyondy DO, Cundall PA. 2004 A bonded-particle model for rock. Int. J. Rock Mech. Min. Sci. **41**, 1329-1364. (10.1016/j.ijrmms.2004.09.011)

[41] Ji CM, Zhang ZH, Ye DH. 2008 The influence of the disk cutter space on rock's jump break coefficients. J. Basic Sci. Eng. **16**, 255-263.

[42] Kruyt NP. 2012 Micromechanical study of fabric evolution in quasi-static deformation of granular materials. Mech. Mater. **44**, 120-129. (10.1016/j.mechmat.2011.07.008)

[43] Cambou B, Magoariec H, Nguyen N-S. 2016 Texture, stress and strain at the meso-scale. In Granular materials at meso-scale towards a change of scale approach, pp. 49-80. London, UK: ISTE Press.

[44] Geng Q, He F, Lu Z, Liu X, Wang X, Ye M. 2022 Geometry evolution of mesoscopic mechanical structures during the rock fragmentation process induced by tunnel boring machine (TBM) cutters. *FigShare*. 10.1098/rsos.211630 PMC8790356 35116164

